# Achieving the Balance between ROS and Antioxidants: When to Use the Synthetic Antioxidants

**DOI:** 10.1155/2013/956792

**Published:** 2013-04-29

**Authors:** Borut Poljsak, Dušan Šuput, Irina Milisav

**Affiliations:** ^1^University of Ljubljana, Laboratory of Oxidative Stress Research, Faculty of Health Sciences, Zdravstvena Pot 5, SI-1000 Ljubljana, Slovenia; ^2^University of Ljubljana, Faculty of Medicine, Institute of Pathophysiology, Zaloska 4, SI-1000 Ljubljana, Slovenia

## Abstract

Free radical damage is linked to formation of many degenerative diseases, including cancer, cardiovascular disease, cataracts, and aging. Excessive reactive oxygen species (ROS) formation can induce oxidative stress, leading to cell damage that can culminate in cell death. Therefore, cells have antioxidant networks to scavenge excessively produced ROS. The balance between the production and scavenging of ROS leads to homeostasis in general; however, the balance is somehow shifted towards the formation of free radicals, which results in accumulated cell damage in time. Antioxidants can attenuate the damaging effects of ROS *in vitro* and delay many events that contribute to cellular aging. The use of multivitamin/mineral supplements (MVMs) has grown rapidly over the past decades. Some recent studies demonstrated no effect of antioxidant therapy; sometimes the intake of antioxidants even increased mortality. Oxidative stress is damaging and beneficial for the organism, as some ROS are signaling molecules in cellular signaling pathways. Lowering the levels of oxidative stress by antioxidant supplements is not beneficial in such cases. The balance between ROS and antioxidants is optimal, as both extremes, oxidative and antioxidative stress, are damaging. Therefore, there is a need for accurate determination of individual's oxidative stress levels before prescribing the supplement antioxidants.

## 1. Introduction 

**Figure 1:**
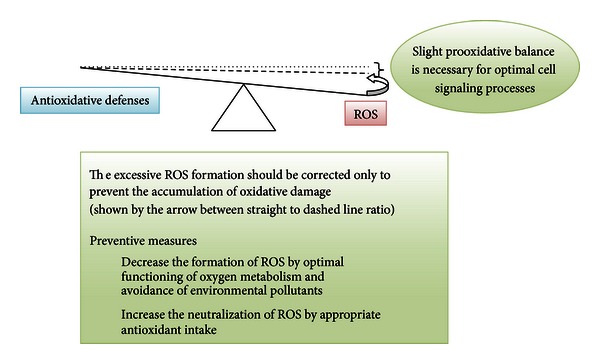
Model antioxidative/oxidative balance of an adult person—the balance is slightly moved towards the increased ROS production (dashed line). The physiological balance is represented by the dashed line and not the dotted line (geometrical balance), since slight pro-oxidative balance is necessary for optimal immune system and cell signaling processes.

**Figure 2:**
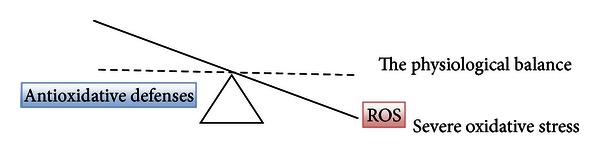
Oxidative stress due to severe disturbance between ROS formation and antioxidative defenses (the physiological balance is represented by the dashed line).

Free radicals are reactive chemicals with an unpaired electron in an outer orbit [1]. Reactive oxygen species (ROS) comprise both free radical and nonfree radical oxygen containing molecules such as hydrogen peroxide (H_2_O_2_), superoxide (O_2_^∙−^), singlet oxygen (1/2O_2_), and the hydroxyl radical (^∙^OH). There are also reactive nitrogen, iron, copper, and sulfur species [1, 2] which could attribute to increased ROS formation and oxidative stress and impair the redox balance. No matter how careful we are, we cannot avoid endogenous and exogenous free radical formation due to normal metabolism and exposure to environmental oxidants [3]. Free radicals are produced when our cells create energy from food and oxygen and when we are exposed to microbial infections, extensive exercise, or pollutants/toxins such as cigarette smoke, alcohol, ionizing and UV radiations, pesticides, and ozone. The most important endogenous sources of oxidizing agents contributing to aging are mitochondrial: electron transport chain and nitric oxide synthase reaction. Nonmitochondrial sources of free radicals are Fenton's reaction, microsomal cytochrome P_450_ enzymes, peroxisomal beta-oxidation, and respiratory burst of phagocytic cells [4]. It has been shown that oxidative stress is involved in over 100 diseases, as their cause or consequence [2, 5]. Oxidative stress was first defined by Sies [6] as “a disturbance in the prooxidant to antioxidant balance in favor of the former, leading to potential damage” (Figures 1 and 2). Oxidative stress can be defined as an excessive amount of ROS, which is the net result of an imbalance between production and destruction of ROS (the latter is regulated by antioxidant defences). Oxidative stress is a consequence of an increased generation of free radicals and/or reduced physiological activity of antioxidant defenses against free radicals. All forms of life maintain a reducing environment within their cells. This reducing environment is preserved by enzymes that maintain the reduced state through a constant input of metabolic energy. Disturbances in this normal redox state can cause toxic effects through the production of peroxides and free radicals that damage all components of the cell. Severe oxidative stress can cause cell death. The degree of oxidative stress experienced by the cell will be a function of the activity of ROS generating reactions and the activity of the ROS scavenging system. In physiological conditions, the balance between prooxidant and antioxidant substances is kept slightly in favor of prooxidant products, thus favoring a mild oxidative stress (Figure 1) [7].

## 2. Beneficial Use of Antioxidants

A biological antioxidant has been defined as any substance that is present at low concentrations compared to an oxidizable substrate and significantly delays or prevents the oxidation of that substrate [8]. An ideal antioxidant should be readily absorbed by body and should prevent or quench free radical formation or chelate redox metals at physiologically relevant levels. It should work in aqueous and/or membrane domains and effect gene expression in a positive way [9]. Cellular redox homeostasis is carefully maintained by an elaborate endogenous antioxidant defense system, which includes endogenous antioxidant enzymes such as superoxide dismutase (SOD), catalase, glutathione peroxidase (GPx), glutathione (GSH), proteins, and low-molecular-weight scavengers, like uric acid, coenzyme Q, and lipoic acid. The human antioxidant defense is complex and must minimize the levels of ROS while allowing useful roles of ROS to perform cell signaling and redox regulation [10]. 

Generation of ROS and the activity of antioxidant defense appear more or less balanced *in vivo*. In fact, as already mentioned, the balance may be slightly tipped in favor of the ROS so that there is continuous ROS formation and low-level oxidative damage in the human body (Figure 1). This creates a need for a second category of endogenous antioxidant defense system, which removes or repairs damaged biomolecules before they accumulate and result in altered cell metabolism and permanent damage [11]. Oxidatively damaged nucleic acids are repaired by specific enzymes, oxidized proteins are removed by proteolytic systems, and oxidized lipids are repaired by phospholipases, peroxidases, and acyl transferases [11]. It seems that failures of some or all of the repair systems contribute more to aging and age-related diseases than moderate oscillations in antioxidants and ROS formation [12–14]. Many of the essential maintenance repair systems become deficient in senescent cells, cell damage accumulates, for example, lysosomal accumulation of lipofuscin [15, 16]. Age-related oxidative changes are most prominent in nonproliferating cells, such as neurons and cardiac myocytes because there is no “dilution” of damaged structures through cell division [17]. Besides, Dröge and Schipper [18] and Bokov et al. [19] proposed that general signalling failure with aging could be due to insufficient reactive species or wrong reactive species production although it is known that oxidative damage increases with age in a variety of tissues and animal models [19]. 

The effects of increased fruit and vegetable intake are associated with lowered parameters of cell damage *in vitro*, for example, lower oxidative stress, DNA damage, malignant transformation rate, and so forth; epidemiologically they seem to result in lowered incidence of certain types of cancer and degenerative diseases, such as ischemic heart disease and cataract [20–25]. On the other hand, increased or prolonged free radical action can overwhelm ROS defense mechanisms, contributing to development of diseases and aging. Since oxidative damage of our cells increases with age, the increased intake of exogenous antioxidants from fruit and vegetables may support the endogenous antioxidative defense. The antioxidants, like vitamin C and E, carotenoids, and polyphenols (e.g., flavonoids), are presently considered to be the main exogenous antioxidants. Clinical studies imply that eating a diet rich in fruits, vegetables, whole grains, legumes, and omega-3 fatty acids can help humans in disease prevention [26].

## 3. Use of Synthetic Antioxidants: Their Control and Safety

A dietary supplement, also known as a food or nutritional supplement, is a preparation intended to provide nutrients such as vitamins, minerals, fibres, fatty, or amino acids that are either missing or not consumed in sufficient amounts in person's diet. Surveys indicate that more than half of the US adult population uses food supplements, many of which contain antioxidants, such as vitamin A (retinoids, carotenes), vitamins C and E (tocopherols), lycopene, lutein, ubiquinone, glutathione, polyphenols (flavonoids), resveratrol, and N-acetylcysteine. In the USA, food supplements represent a market of over $7 billion/year [27] and exceed $30 billion worldwide [28].

In the United States of America (and in many other countries), the dietary supplement or dietary ingredient manufacturer is responsible for ensuring that a dietary supplement or ingredient is safe before it is marketed under the Dietary Supplement Health and Education Act of 1994 [29]. Generally, manufacturers neither need to register their products with Food and Drug Administration (FDA) nor get FDA approval before producing or selling dietary supplements. FDA is responsible for taking action against any unsafe dietary supplement product after it reaches the market. Manufacturers must make sure that product label information is truthful and not misleading and are required to submit to FDA all serious adverse event reports associated with use of the dietary supplement in the United States. In contrast, the substances used as drugs must undergo clinical studies to determine their effectiveness, safety, possible interactions with other substances, and appropriate dosages before entering the market [30]. FDA independently reviews company's data and proposed labeling and, if health benefits outweigh its known risks, it approves the drug for sale.

**Figure 3:**
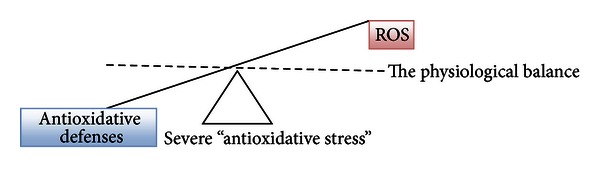
Severe disturbance between antioxidative defenses and ROS leads to a state of increased “antioxidative stress.”

The inappropriate use of dietary supplements may lead to “antioxidative stress.” This term was used for the first time by Dundar and Aslan [31] for description of the negative effects of antioxidants; it is discussed also by recent publication by Poljsak and Milisav [32]. Both “antioxidative” and oxidative stresses leading to the antioxidative imbalance can be damaging for the organism and can result in cancerogenesis [33] and aging (Figures 2 and 3). There are a growing number of clinical trials in which individuals received one or more synthetic antioxidants that fail to demonstrate beneficial effects of antioxidant supplementation. Some even implied that antioxidant therapy had no effect and even could increase mortality [34–46]. Ristow et al. [47] reported that nutritive antioxidants abolished the life extension by inhibiting a process called mitohormesis. Results of clinical trials on exogenous antioxidants intake are thus conflicting and contradictory. There seem to be homeostatic mechanisms in cells that govern the total antioxidant activity. Modifying the levels of one antioxidant causes compensatory changes in the levels of others, while the overall antioxidant capacity remains unaffected. The intake of only one antioxidant may thus alter the complex system of endogenous antioxidative defence of cells or alter the cell apoptosis pathways [48]. Dosing cells with exogenous antioxidants may decrease the rate of synthesis or uptake of endogenous antioxidants, so that the total “cell antioxidant potential” remains unaltered. Cutler [49, 50] introduced “The oxidative stress compensation model” to explain why dietary supplements of antioxidants have minimal effect on longevity. He explains that most humans are able to maintain their set point of oxidative stress even if they consume additional antioxidant supplements; in other words, there is no further decrease in oxidative stress [49, 50].

## 4. Importance of the Balance

The production of free radicals increases with age [51], while some of the endogenous defense mechanisms decrease [52]. This imbalance leads to progressive damage of cellular structures, presumably resulting in the aging phenotype [53, 54]. 

The antioxidant defense system must thus minimize the levels of most harmful ROS on one side while still permit enough ROS to remain for their useful purposes (e.g., cell signaling and redox regulation). Cells usually tolerate such mild oxidative stress; this stress can even upregulate cellular repair processes and other protective systems (e.g., chaperones). 

## 5. “Antioxidative Stress” Influences Cell Signaling and Redox Regulation

The beneficial physiological cellular use of ROS is now being demonstrated in different fields, including intracellular signaling and redox regulation. It is well documented that low levels of ROS are signaling molecules, modulating cell proliferation [55], apoptosis [56, 57], and gene expression through activation of transcription factors [58], like NF-kappa-B and hypoxia-inducible-factor-1*α* (HIF) [59]. The inducers of NF-kappa-B include also tumor necrosis factor alpha (TNF*α*) and interleukin 1-beta (IL-1*β*) [60, 61]. ROS can act as signaling intermediates for cytokines, including IL-1 and TNF*α* [62–64]. These proinflammatory cytokines, tumor necrosis factor (TNF)-*α*, interleukin-1*β* (IL-1*β*), and interferon-*γ* (IFN-*γ*), can additionally increase oxidative stress in humans [65], inducing production of ROS [64, 66]. ROS also have a role in vascular cell signaling processes including activation of eNOS [67] and stimulation of cell growth and migration [68] through modulation of intracellular calcium [69] and activation of transcription factors such as NF-kappa-B [70] and protein kinases including ERK, p38MAPK, and Akt [71, 72]. ROS signaling is thus integrated into many cellular pathways, including but not limited to (1) proliferation and survival pathways through MAP kinases, PI3 kinase, PTEN, and protein tyrosine phosphatases; (2) ROS homeostasis and antioxidant gene regulation through Ref-1, Nrf-2, thioredoxin, and so forth; (3) aging through p66Shc; (4) DNA damage response through ATM kinase; this may lead to inhibition of mTORC1 resulting in suppression of protein synthesis and activation of autophagy; (5) iron homeostasis through iron-regulatory proteins (IRP) and iron-responsive elements (IRE), and so forth [73].

The production of O_2_^∙−^ and H_2_O_2_ by activated phagocytes is the classic example of the deliberate metabolic generation of ROS for useful purposes [74]. H_2_O_2_ is recognized as an ubiquitous intracellular messenger [75–78]. Moderate amounts of mitochondrial superoxide and hydrogen peroxide have important roles in a range of cellular signaling processes and can activate signaling pathways that promote cell survival and disease resistance due to hormesis [79–81]. Generation of O_2_^∙−^, HOCl, and H_2_O_2_ by phagocytes is important for defense against various bacterial and fungal strains [82]. O_2_^∙−^ is produced also by several cell types other than phagocytes, including lymphocytes and fibroblasts [82]. As ROS are important in signal transduction, there seem to be no great reserve of antioxidant defenses in mammals [83]. 

## 6. Imbalance between ROS and Antioxidants

### 6.1. Increased Oxidative Stress

The causes of increased ROS production include endogenous reasons (inflammation, elevation in O_2_ concentration, and increased mitochondrial leakage) and exogenous (environmental pollution, strenuous exercise, smoking, nutrition, chronic inflammation, psychological and emotional stress, and others) [3, 32, 79]. Causes of decreased antioxidant defenses include reduced activity of endogenous antioxidative enzymes and reduced intake or absorption of antioxidants from food.

### 6.2. Increased “Antioxidative Stress”

Inappropriate antioxidative intake may cause increased “antioxidative stress.” Antioxidants can neutralize ROS and decrease oxidative stress; however, this is not always beneficial with respect to the development of a disease and its progression (e.g., cancer) or for delaying aging [32] since antioxidants cannot distinguish among the radicals with a beneficial physiological role and those that cause oxidative damage to biomolecules.

Individuals who overdose antioxidant supplements could enter the status of “antioxidative” stress (Figure 3). If administration of antioxidant supplements decreases the level of free radicals, it may interfere with the immune system to fight bacteria and essential defensive mechanisms for removal of damaged cells, including those that are precancerous and cancerous [84]. Thus, antioxidant supplement overtake may cause harm [35, 36, 56, 85, 86]. When large amounts of antioxidant nutrients are taken, they can also act as prooxidants by increasing oxidative stress [87, 88]. Pro- and antioxidant effects of antioxidants (e.g., vitamin C) are dose dependent, and thus, more is not necessarily better. Our diets typically contain safe levels of vitamins; therefore, high-level antioxidant supplements may upset this important physiological balance between the ROS formation and neutralization. 

**Figure 4:**
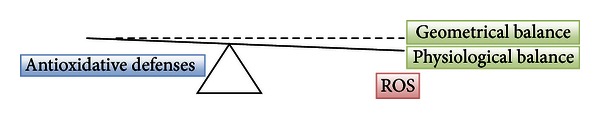
The optimal situation: the physiological balance between the ROS production and antioxidative defenses prevents the accumulation of damage by ROS and enables enough ROS for signaling. Enzymatic and nonenzymatic antioxidants can neutralize ROS and RNS and decrease oxidative stress and restore the balance.

The amount of oxidized macromolecules in the cell is the sum of the rate of their formation subtracted by the rate of repair processes. The imposed oxidative damage potential is opposed by the antioxidant defense capacity of the system (Figure 4). In reality, the oxidative damage potential is greater, and thus there is a constant small amount of toxic free radical formation, which escapes the defense of the cell. A certain amount of oxidized proteins and nucleic acids exists in cells at all times; this reflects the oxidative events. Decreased compensation of oxidative stress and insufficient repair, in turn, accelerate aging, which consequently leads to further decline of cellular energy levels [89, 90]. Mechanisms that protect cells from oxidative stress (e.g., endogenous antioxidants, DNA repair processes) are consuming significant amounts of energy when being activated in all compartments of the cell for prolonged time. It may require too much energy to prevent all oxidative damage throughout the life of an organism. Kowald and Kirkwood predicted that virtual immortality might be achieved if 55% of the total energy of the simulated cell were devoted to repair and/or prevention of free radical and oxidative damage on the quantitative MARS model (mitochondria, aberrant proteins, radicals, and scavengers) [91, 92]. It is the compromise to allocate suboptimal amounts of energy to cell repair systems, with a consequence of gradual deterioration of the body structures with age [93]. Paradoxically, the efficiency of defense and repair may be enhanced also after the exposure to ROS, since the expression of many DNA repair enzymes is upregulated during the oxidative stress [94–96]. Finkel and Holbrook [97] stated that the best strategy to enhance endogenous antioxidant levels may actually be oxidative stress itself, based on the classical physiological concept of hormesis [98]. This is in agreement with a recent Halliwell's proposal that stimulating the increase in levels of endogenous antioxidants by some prooxidants may be more effective than consuming additional dietary antioxidants [10]. Many well-established components of the heart-healthy lifestyle are prooxidant, including the polyunsaturated fat, exercise, and moderate alcohol consumption [99].

## 7. The Importance of Determination of the Oxidative/Antioxidative Status *In Vivo*

In order to determine the oxidative stress, both, the ROS formation as well as the antioxidative defense potential should be measured; for example, low antioxidant amount is not problematic when the ROS levels are low.

### 7.1. Determination of ROS

**Scheme 1:**
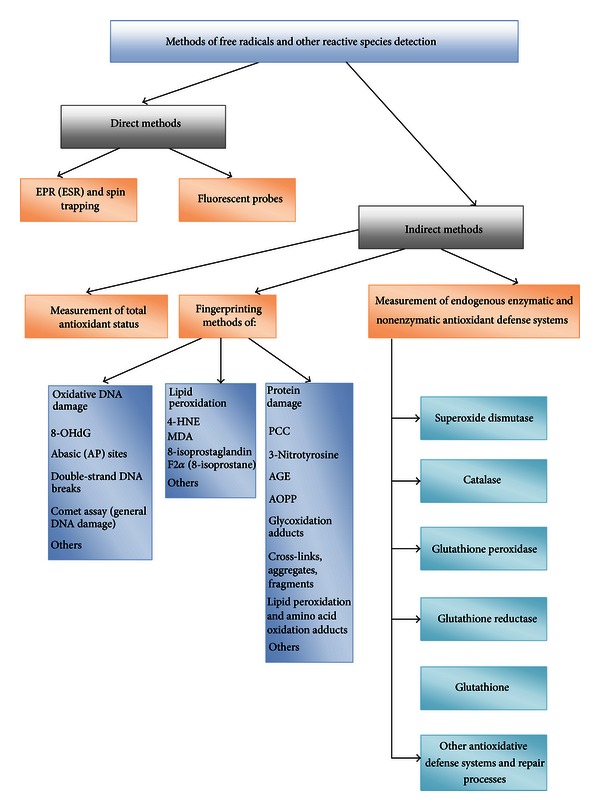
Methods of oxidative stress determination. (8-OHG) = 8-hydroxyguanosine; 4-HNE = 4-Hydroxynonenal; MDA = malondialdehyde; PCC = protein carbonyl content; AGE = advanced glycation end products; AOPP = advanced oxidation protein products.

Free radicals have a very short half-life, which makes them hard to measure in the laboratory. Nevertheless, multiple methods of oxidative stress measurement are available today, each with their own advantages and disadvantages (see review [101]). Many approaches are possible: identification of free radicals, either directly by paramagnetic electron resonance (electron spin resonance, ESR), or indirectly by identifying some more stable intermediates: evaluation of the traces of radical attack on biological molecules by high performance liquid chromatography, gas-liquid chromatography, colorimetric tests. The measurement of antioxidant status can be estimated by colorimetric, immune, or enzymatic methods [100] (Scheme 1). The direct ROS detection methods measure superoxide, H_2_O_2_, ^∙^OH. These are very reactive species and their quantitation is difficult. *In vivo* ESR is relatively insensitive and requires steady-state concentrations of free radicals in the micromolar range, which limits its use for measuring ROS in patients. ESR can be applied only through the technique of spin trapping for *in vivo *samples. Although it seems that toxicity is not a serious problem for most traps, there are no effective spin traps to be administered to humans. Indirect methods are used in order to overcome these problems. Indirect methods usually measure the changes in endogenous antioxidant defense systems or measure the ROS-induced damage of cellular components [101]. Measuring the damage caused by ROS instead of direct measuring of ROS seems logical, since it is the damage caused by ROS that is important rather than the total amount of generated ROS. Methods have been developed to detect and quantify oxidative damage to proteins, lipids, and DNA. The principle behind fingerprinting methods is to measure products of damage by ROS, that is, to measure not the species themselves but the damage that they cause. Of course, the end-products must be specific markers of oxidative damage [8]. According to Miwa et al. [102], a good marker of oxidative damage must increase by oxidative stress (i.e., upon the treatment with, e.g., paraquat, diquat, ionizing radiation, hyperoxia), and it must remain unchanged in the absence of the oxidative event.

### 7.2. Determination of Antioxidant Status

There is a growing interest to measure antioxidant status for clinical assessment [103]. Cellular protection against unwanted oxidation is achieved mainly by enzymes, such as superoxide dismutase (SOD), catalase, and glutathione peroxidase, whereas the nonenzymatic antioxidants are playing the major role in the plasma. Radical-scavenging antioxidants are consumed during the reactions with ROS, and antioxidant status could be used indirectly to assess the free radical activity. One approach is to measure individual antioxidants (e.g., ascorbate, *α*-tocopherol, urate) in blood, plasma, or tissue homogenates. All of the individual molecules that are currently recognized as antioxidants should be measured [103]. However, this approach has several shortcomings: (1) it is time consuming, expensive, and technically demanding, (2) it may not detect the synergistic effects between the antioxidants, and (3) it may not account for the influence of presently unknown antioxidant substances. The other approach is to measure the total antioxidant capacity or activity by subjecting the samples to controlled oxidative stress conditions and measuring either the rate of oxidation or how long it takes for oxidation to occur. Determination of antioxidative potential per se is not sufficient, since it is difficult to establish how the individual antioxidants work: by preventing the formation of ROS, by scavenging free radicals, by inducing the signaling pathways, or by repairing the oxidative damage. Additionally, antioxidative status differs significantly between the individuals and between the laboratory methods used in humans [101]. Typical oxidative stress status of an individual is not established so far [104]. There are no reference values on the optimal levels of antioxidants in urine, blood, or even intracellularly. Additionally, several free radicals cannot cross cell membranes due to their charge, or they are so short-lived that their diffusion is negligible. As such they cannot enter the blood from an affected region or organ. As there is no direct correlation between the oxidative stress markers in blood and their levels within the cells measuring the blood samples may be misleading. Besides, unknown are the amounts and combinations of antioxidants needed for the beneficial effect *in vivo*. The improved methodology for determining the oxidative stress levels in humans may overcome at least some of these drawbacks.

**Scheme 2:**
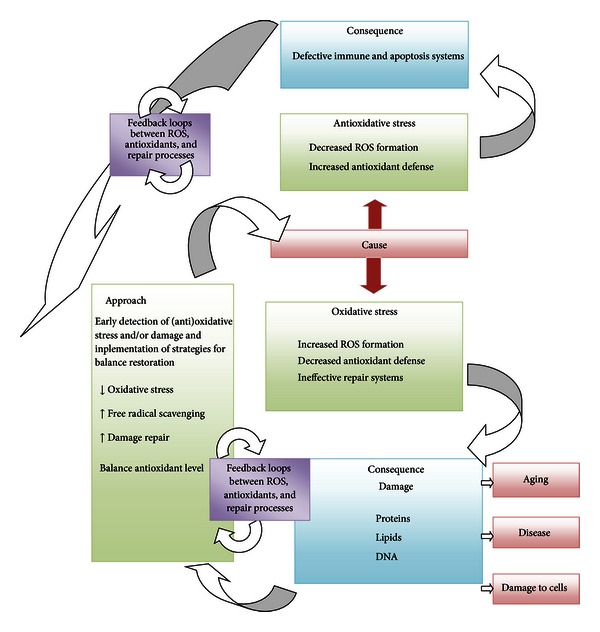
Oxidative and “antioxidative” stress: causes, consequences and methods for its control.

Long-term effects of oxidative stress will occur if antioxidant status is low and levels of free radicals are high. No specific clinical symptoms or clinical signs are associated with oxidative stress during the early stages of imbalance. Therefore, the oxidative stress is not diagnosed until there is an unavoidable damage and the consequences manifest as a sign of a disease that could last for decades. Thus, oxidative stress should be recognized and the oxidative imbalance should be ameliorated in order to prevent or postpone the free radical-related disease development and premature aging [32]. In practice, it is difficult to determine all types of ROS within the cells or cellular compartments, as well as the overall antioxidative protection and repair of cells and organs at any specific time. Increased oxidative stress could result from increased ROS production or from decreased antioxidative defences. Thus, increased ROS damage could be the result of (a) increased ROS formation, (b) decreased antioxidative defence, or/and (c) altered damage repair (Scheme 2). Since none of the biomarkers can predict the disease development as the consequence of the prolonged oxidative stress [105], it is important to use many methods for detection and quantification of oxidative stress whenever possible in order to enhance their validity, as each method measures different parameters and has inherent limitations. No single method can measure the oxidative stress or its subsequent damage *in vivo* accurately at present. Additionally, the living organisms are complex and ever changing systems and therefore any determination of the oxidative stress levels reflect the temporary state that may change considerably over time. 

Presently, the use of supplemental antioxidants could be advised only in cases of well-known conditions, where the depletion of antioxidants is known and can be predicted. Daily use of synthetic supplements has not been proven as beneficial, and excessive use may be harmful. Balanced food still seems to be the best option. 

## 8. Discussion

A complex mix of substances in fruits and vegetables may contribute to improved cardiovascular health and decreased incidence of cancer in individuals who consume more of these foods [22, 23]. Even in elderly subjects a higher daily intake of fruits and vegetables is associated with an improved antioxidant status compared to subjects consuming diets poor in fruits and vegetables [106]. Contrary, many clinical trials in which individuals received one or more synthetic antioxidants failed to prove their benefits. None of the major clinical trials using mortality or morbidity as the end point has found positive effects of supplementation with antioxidants such as vitamin C, vitamin E, or *β*-carotene. Some recent studies showed that antioxidant therapy had no effect and even increased the mortality [34–38, 107]. The intake of only one antioxidant could alter the endogenous antioxidative defense of cells, modify cell death rates, or decrease the synthesis of endogenous antioxidants. We have to realize that the use of synthetic vitamin supplements is not the alternative to the regular consumption of fruits and vegetables. Cutler explains that most humans are able to maintain their set points of oxidative stress regardless of additional antioxidant supplementation through diet [49, 50]. In contrast, antioxidant supplements do appear to be effective in lowering an individual's oxidative stress if his/her initial oxidative stress is above normal or above his/her set point of regulation [49, 50]. Thus, the antioxidant supplements may help the organism to correct the elevated levels of oxidative stress that cannot be controlled by the endogenous antioxidants. There is also a problem of dosing the synthetic antioxidants; for example, there are claims that RDA (recommended daily allowance) levels of vitamin C and E are too low to prevent the oxidative stress. On the other hand, many consumers ingest high amounts of supplements with the antioxidant potential, which may lead to prooxidant effects or to “antioxidative stress” [32]. Therefore, there is a need to determine the individual's oxidative stress level before administering the supplement therapy. However, the reference values for typical oxidative stress status of an individual are not established so far and oxidative stress is difficult and expensive to measure [104]. 

## 9. Conclusion

*In vitro* and *in vivo* studies imply that antioxidant nutrients and related bioactive compounds from fruits and vegetables can protect us from oxidative stress. Synthetic antioxidants as dietary supplements may prevent some ROS-induced damage in conditions of elevated oxidative stress during elevated environmental oxidant exposure or at weaken endogenous oxidative stress responses of an aged organism. On the other hand, the presented evidence implies that synthetic antioxidant supplements cannot offer appropriate or total protection against oxidative stress and damage in “normal” situations and that the use of antioxidants to prevent disease or aging is controversial in situations of “normal” oxidative stress. 

At the moment, it is difficult to evaluate the oxidative stress of the organism also because different criteria of oxidative stress do not correlate with each other. Since there is no universal “scale” of oxidative stress, the future challenge(s) are in determination of total antioxidants and oxidative stress levels in different body fluids (urine, saliva, blood, and cytosol). Further, detection of the increased levels of oxidative stress biomarker in the body fluid does not mean necessarily that the cells of the specific organ or tissue are under oxidative stress. Besides, it is not possible for highly reactive free radical produced within a tissue with a lifetime of microseconds to diffuse into the blood to be detected at the distant site. The researcher is thus limited to determination of secondary products in human body fluids distant from the locus of the ROS production [108]. With indirect oxidative stress markers, the person may be considered being under oxidative stress according to a given criterion but not to another. Therefore, there is an urgent need to compare and standardize the various methods for assessing the oxidative state of biological systems, to establish the universal scale of oxidative stress, and to provide age and gender specific tables of “normal values” for each body fluid. Until these are established, it is prudent to estimate the oxidative stress by combining different methods and biomarkers. 

The key to the future success of dietary antioxidant supplementation may be in the fine tuning of the suppression of oxidative damage without disruption of the well-integrated antioxidant defense networks. The selective enhancement of the defense system could be a major strategy for a successful intervention by antioxidant administration [109].
